# Transitioning from climate ambitions to climate actions through public health policy initiatives

**DOI:** 10.1097/EE9.0000000000000373

**Published:** 2025-03-06

**Authors:** George D. Thurston, Zorana J. Andersen, Kristine Belesova, Kevin R. Cromar, Kristie L. Ebi, Christina Lumsden, Audrey de Nazelle, Mark Nieuwenhuijsen, Agnes Soares da Silva, Oriol Teixidó, Mary B. Rice

**Affiliations:** a Department of Medicine, New York University Grossman School of Medicine, New York, New York; b Department of Public Health, University of Copenhagen, Copenhagen, Denmark; c Imperial College School of Public Health, London, United Kingdom; d New York University, Marron Institute of Urban Management, New York, New York; e University of Washington, Center for Health and the Global Environment, Seattle, Washington; f C40 Cities, London, United Kingdom; g Imperial College, Centre for Environmental Policy, London, United Kingdom; h Barcelona Institute for Global Health: Barcelona, Spain; i Department of Environmental and Worker Health Surveillance, Ministry of Health, Brasilia, Brazil; j Ricardo plc., Abu Dhabi, United Arab Emirates; k Center for Climate, Health and the Global Environment (C-CHANGE), Harvard Chan School of Public Health, Boston, Massachusetts; l Division of Pulmonary, Sleep and Critical Care Medicine, Beth Israel Deaconess Medical Center, Harvard Medical School, Boston, Massachusetts.

**Keywords:** public health policy implementation, climate mitigation action, motivation, fossil fuel combustion transition, civic engagement

## Abstract

Policies to implement climate-forcing pollution emission reductions have often been stymied by economic and political divisiveness. However, certain uncontested nonregret public health policies that also carry climate-forcing cobenefits with them could provide more achievable policy pathways to accelerate the implementation of climate mitigation. An International Society for Environmental Epidemiology Policy Committee endorsed pre-28th Conference of the Parties climate meeting workshop brought together experts on environment, diet, civic planning, and health to review current understanding of public health policy approaches that provide climate change mitigation cobenefits by also reducing greenhouse gas emissions. Promising public health policy areas identified as also providing climate mitigation cobenefits included: improving air quality through stronger regulation of harmful combustion-related air pollutants, advancing healthier plant-based public food procurement programs, promoting more sustainable transport options, developing healthier infrastructure (e.g., combustion-free buildings), and reducing the use of climate forcing substances in healthcare. It is concluded that cities, states, and nations, when aided by involved health professionals, can advance many practical public health, diet, and civic planning policies to improve health and well-being that will also serve to translate climate mitigation ambitions into action.

**Key conclusions and recommendations**

This ISEE-endorsed workshop titled “Climate Action and Health” served as a platform for fostering productive discussions and facilitating the exchange of ideas, notably:

- Many public health policy measures can also achieve climate-forcing pollution reductions;
- Unlike most proposed climate-forcing pollution policies (e.g., a new carbon tax), many climate-friendly public health policies have existing regulations and policy guidelines that can be readily supported and implemented to achieve both public health and climate progress, and;
- Further civic engagement is needed by scientists and health professionals to advance climate-friendly public health measures.

**What this study adds**

Policies to implement climate-forcing pollution emission reductions have too often been stymied by political divisiveness and a lack of popular motivation for immediate climate change mitigative actions to prevent a problem that seems largely distant in space and time. This collaborative effort identified an additional pathway that can engender climate action: leveraging existing public health policy mechanisms to promote healthy actions that also carry with them climate mitigative cobenefits. Examples are provided by which health professionals, planners, decision-makers, and patient organizations can civically engage to implement health policy pathways that are also climate and sustainability friendly.

## Introduction

A cross-disciplinary group met at the New York University Research Institute in Abu Dhabi, UAE, immediately prior to the 28^th^ United Nations Conference of the Parties climate meeting (held in December 2023 at Dubai, United Arab Emirates), to identify opportunities where public health and public policy initiatives can also bring climate mitigation cobenefits. Participants presented the current state of the science in their respective fields, based on their expertise and a review of the latest research available on their specific topics. This is a consensus document, rather than a formal systematic examination of all the evidence. A writing committee summarized workshop findings, which participants reviewed for an accurate reflection of the proceedings.

This 2-day 27–28 November 2023 preconference focused on the identification of areas of public health policy intersections with climate mitigation through several key questions:

- What are climate change mitigation-friendly approaches to reach societal health goals?
- What are regulatory and guideline pathways to achieve climate-friendly health policies?
- What are the mechanisms by which health professionals, planners, decision-maker, and patient organizations can act to help implement the most climate and sustainability-friendly health policies?

This report on the Workshop is structured to first briefly summarize the information reported by the various speakers, followed by assessments of how the workshop content addressed the questions.

## Background

Climate change, if not strongly mitigated, will likely become the greatest environmental and health challenge of the 21^st^ Century. While nations around the world have pledged climate ambitions as part of the United Nations Conference of the Parties process, the practical pathways to motivating and achieving climate action are less well defined, and actual progress has been inadequate. Moreover, policies aimed directly at implementing climate-forcing pollution emission reductions have often been stymied by political divisions. However, the human health cobenefits of many mitigative actions can help motivate local and immediate climate-forcing action.^1^ Indeed, the Lancet Health and Climate Change Commission has declared that the climate mitigative actions we take can have the potential to provide the greatest public health opportunity of the 21^st^ Century.^2^ These actions can include changes in our societal diets, lifestyles (e.g., active transportation), sustainable development, and greener (and cleaner) energy choices, resulting in improved cardiovascular and other human health cobenefits.^3–7^ The extent of some of these health cobenefits of climate forcing mitigation have been tracked on annual basis at the global scale by the Lancet Countdown on Climate Change and Health since 2016 in its working group indicators.^8^ Conversely, more politically viable public health and civic planning policies that also carry climate-forcing cobenefits with them could, at the same time, provide pathways to accelerate the implementation of climate mitigation progress. The overall aim of this workshop was therefore to succinctly identify and summarize knowledge of viable (nonclimate policy) public health and civic actions that can also at the same time engender mitigation of climate change.

## Workshop content

### The public health to climate action paradigm

**Figure 1.**
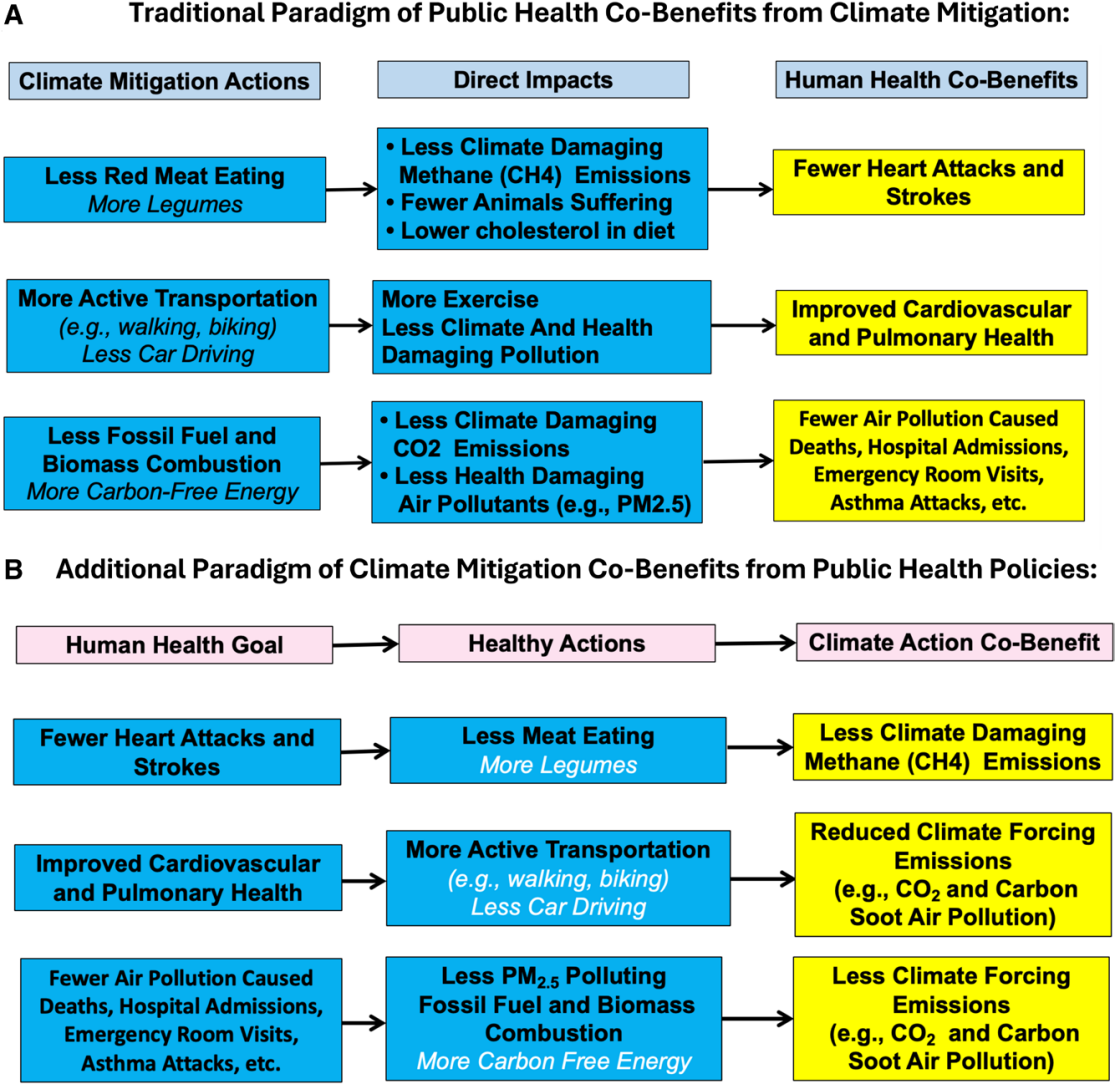
Contrast of climate action approaches.

The workshop was introduced by George Thurston of the Grossman School of Medicine at New York University, who provided an overview of the workshop goals and introduced the core questions considered over the 2-day meeting. A key concept of the meeting introduced is making a transition from a focus on the health cobenefits of policies that address climate change^9,10^ to one that focuses on the climate benefits derivable from more politically achievable public health policies, as contrasted in Figure 1.

An overview of climate mitigation and health benefits was provided by Audrey de Nazelle from Imperial College, based on the recent climate and health publication by International Society for Environmental Epidemiology (ISEE) Policy Committee members.^7^ For example, the report documented the large potential public health benefits of climate action to reduce the sources of climate-forcing pollution, including reducing an estimated 6.7 million deaths globally per year from outdoor air pollution caused by fossil fuel combustion through switching to carbon-free electricity, 3.2 million deaths from indoor combustion air pollution avoidable by eliminating indoor combustion for cooking and home heating, and reducing the 800 thousand avoidable deaths from low levels of physical activity by encouraging active travel (walking, cycling and public transport), as displayed in Figure S1; http://links.lww.com/EE/A332. The introductory talk also highlighted some of the main barriers and facilitators to incorporating health in climate decision-making, suggesting, for example, a role for researchers to produce more policy-relevant systems-based research that will break silos of decision-making and engage society toward transformative action.^5,6^

### Lancet Pathfinder report findings

**Figure 2.**
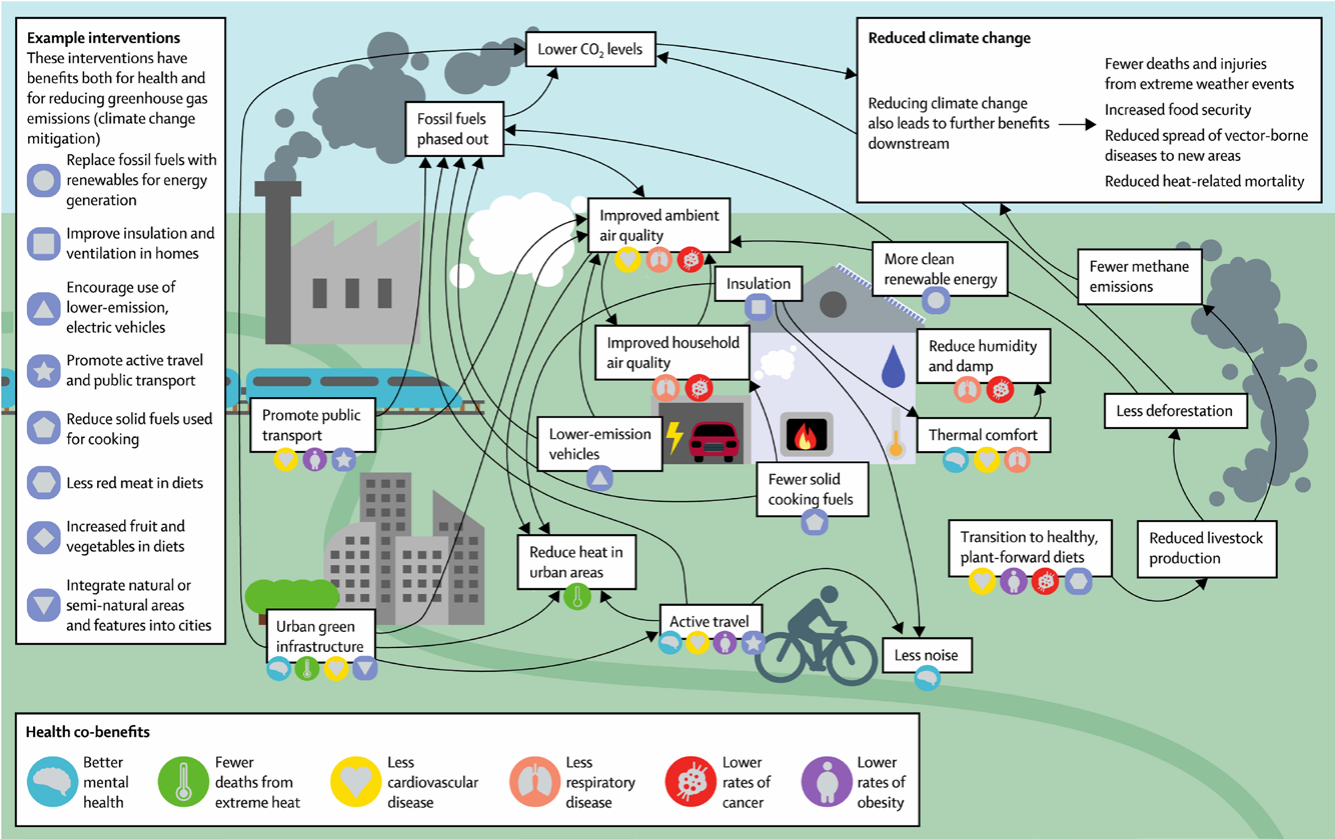
Key pathways and connections between climate mitigation actions and health.^1^ Reprinted with permission from Lancet.

Kristie Ebi from the University of Washington summarized the findings of the most recent Lancet Pathfinder report on climate action and human health benefits.^1^ As summarized in Figure 2, there are many interactions between public health and climate mitigation, providing multiple opportunities for synergisms.^1^ Between 3.5^11^ and 8·7 million,^12^ annual premature deaths are caused globally by air pollution from fossil fuel burning; over 10 million deaths per year could be prevented by shifting to healthier diets^13^; and over 5 million deaths per year linked to physical inactivity,^13,14^ indicating the potentially large human health benefits of those public health policies that also provide climate mitigation cobenefits.

### Urban planning, climate,and health connections

Mark Nieuwenhuijsen summarized the many public health benefits of redesigning our cities to transform them into smart cities that also lower our cities’ carbon footprint. The European Urban Burden of Disease Project was described that is targeting health-preserving urban and transport planning policies to achieve sustainable, livable, and healthy communities. Effective measures include land use changes (e.g., superblocks), reduced car dependency, lowered air pollution, and noise by moving towards public and active transportation, and greening cities. For example, the institution of superblocks in Barcelona has been estimated to achieve a 19% car reduction, an 11.5 ug/m^3^ (24.3%) nitrogen dioxide (NO_2_) reduction, a 2.9 dB noise reduction, a three-fold increase in green space (6.5%–19.6%), and a 20% surface temperature reduction.^15,16^

**Figure 3.**
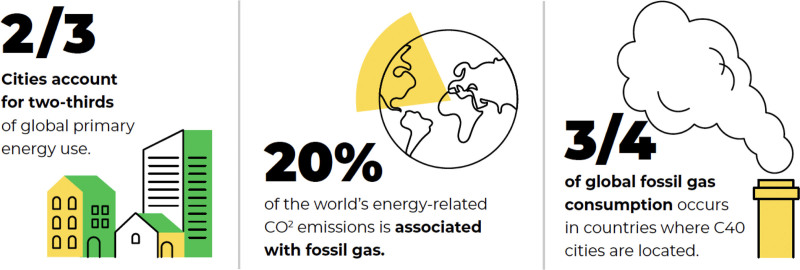
Cities are central to implementation of climate mitigation measures.^17^ Public health- climate connections. Reprinted with permission from C40 Report.

Christina Lumsden from C40 added their experiences in motivating climate action at the city level. Globally, cities account for some two-thirds of primary energy use. C40 is an international network of nearly 100 mayors of the world’s leading cities that are united in action to confront the climate crisis. They represent over 700 million people and a quarter of the global economy. Cities are also responsible for about 75% of global natural resource consumption, making them critical to any solution to climate change. Mayors of C40 cities are committed to using inclusive, science-based, and collaborative approaches to cut their fair share of emissions in half by 2030, help the world limit global heating to 1.5°C, and build healthy, equitable, and resilient communities. An example of the health-based policies being advanced by C40 is a transition away from fossil (natural) gas, which causes both health-threatening air pollution, such as NO_2_, PM_2.5_, and ultrafine particulate matter while, at the same time, releasing climate forcing pollutants, such as methane from supply pipeline leaks, and CO_2_ from fossil gas combustion (Figure 3). Indeed, a recent American Thoracic Society recommended a transition to combustion-free homes and businesses.^17^ The C40 has estimated that a swift, clean energy transition away from fossil gas alone can cumulatively avoid as many as 217,045 premature deaths 198,478 new cases of asthma in children, 17,499 preterm births, 105,045 emergency visits, 127,419, years of disability, 23.6 million sick days in C40 cities by 2035.^18^

Mary Rice of the Harvard School of Medicine discussed the integration of climate-health considerations into routine healthcare as a public health approach that advances climate mitigation behaviors. Healthcare delivery contributes to climate change: the health sector is responsible for almost 5% of global greenhouse gas emissions, and this contribution is as high as 10% in some high-income nations.^19^ The healthcare sector also employs a large commuting workforce that contributes to harmful air pollution from transportation. Physician engagement to address these public health harms can motivate improved energy efficiency and decarbonization of hospital systems. In addition to reducing energy consumption, certain medications emit long-lasting green house gases, such as hydrofluoroalkane propellants emitted by metered dose inhalers used by patients with asthma.^20^ Switching from pressurized metered dose inhalers to dry powder inhalers, when appropriate, can significantly reduce these emissions. Similarly, certain volatile anesthetics have large climate impacts, such as desflurane, which has 3700 times the warming potential of CO_2_.^21^ Engagement from the healthcare industry and health professionals to minimize the public health impact of healthcare delivery can also enable climate mitigation progress in this sector.

**Figure 4.**
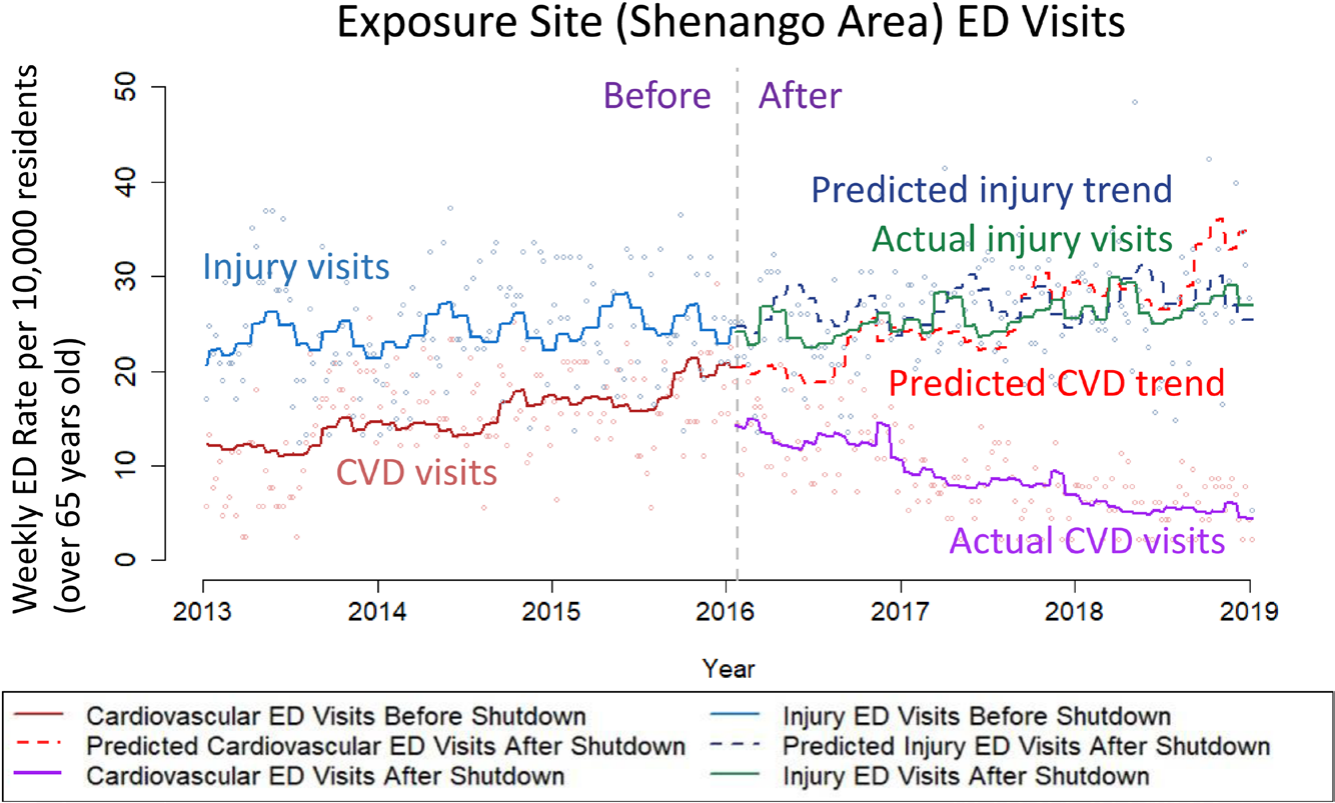
Coal plant closure study demonstrating significant immediate and longer term reductions in cardiovascular hospital visits in nearby communities.^27^

George Thurston of the Grossman School of Medicine at New York University summarized the human health benefits of reducing fine particle air pollution (PM_2.5_) from fossil fuel combustion, which have been found to be among the most toxic types of particulate matter air pollution.^22^ Particulate matter air pollution is well documented as a leading cause of global premature mortality, including that coal-burning derived PM_2.5_ has roughly double the mortality impact per unit mass concentration than found for overall PM_2.5_ mass in general.^23–26^ As shown in Figure 4, a recent coal plant closure in Pittsburgh, PA, dramatically demonstrated the rapid and longer term local cardiovascular benefits of reducing such coal-related air pollution,^26,27^. This included a 42% immediate drop (95% confidence interval: 33%, 51%) in cardiovascular emergency department visits from the preclosure mean in the coking plant neighboring community, and 61% fewer cardiovascular emergency department visits than the predicted trend over the three years after the closure. Along with human health impacting air pollution, this coal plant also emitted some 1.3 million tons/yr of CO_2_ emissions. Clearly, transitioning away from fossil-fuel–based energy sources will provide the world with significant immediate and local human health benefits, as well as help mitigate global climate change.

### Governmental policy—climate connections

**Figure 5.**
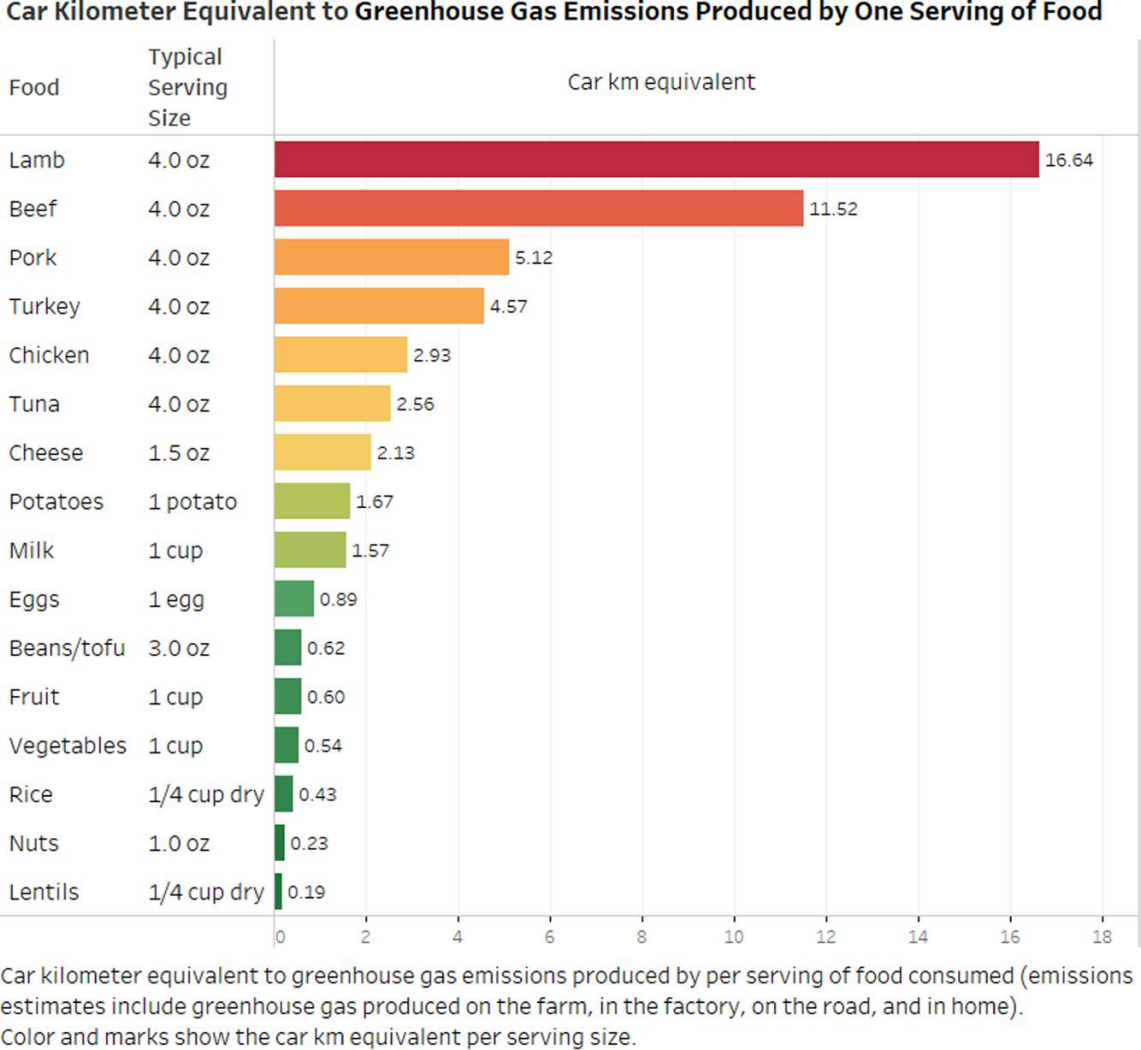
Relative carbon footprint of various foods in equivalent vehicle miles driven per 4 oz. of food (https://www.greeneatz.com/foods-carbon-footprint.html). Reprinted with permission from https://www.greeneatz.com/.

Kristine Belesova from Imperial College London presented the potential to transform public food procurement systems for improved health and sustainability. Many countries operate considerably large public food procurement initiatives that use government purchasing power to meet regular demand for food. For example, one of the world’s largest public food procurement initiatives—Public Distribution System in India—feeds one in 10 people in the world (850 million people). Such systems hold considerable potential to shape food consumption behaviors and norms, as well as food production patterns.^8^ Well-designed interventions targeting these initiatives, such as increased plant-based diets, offer potential for a large-scale food system transformation toward health and sustainability. For example, CO_2_ eq/g of protein from ruminant meats is up to 250 times higher than from legumes (Figure 5). Global food supply transformations such as this could allow the public food sector to meet its healthier diet goals while, at the same time, maximizing climate cobenefits (e.g., from reduced ruminant livestock production). The Pathfinder report found that transitioning to diets that include more plant-based foods and less animal-sourced and processed foods could add 310 life years per 100,000 population annually and would also avoid the release of 30 tons of CO_2_ equivalent emissions per year per 100,00 people.^1^ Other interventions for health and sustainability that can be implemented through public food procurement programs include the introduction of appropriate food production standards, reducing food miles, switching to an electrical vehicle fleet for distribution, more efficient use of energy and switching to clean energy for food storage and preparation, and food waste reduction. Thus, there are climate-friendly government food provision system interventions that offer transformative policy pathways toward both improved health and climate sustainability.

Agnes Soares Da Silva from the Brazil Ministry of Health presented Brazil’s climate-health national mitigation and adaptation plan. As per the Health Sector Plan for Adaptation to Climate Change and Health—Technical-scientific Workshop, 31 October–1 November 2023, the policies will measure and give visibility to populations in situations of vulnerability and health inequities (ethnic/racial, gender, etc.). Data and methods will be provided for situation analysis and prioritization to assess the health risks associated with climate change, assess the effects/impacts that can be measured, and select progress indicators. This plan is expected to prioritize reducing vulnerabilities and increasing the resilience of the national healthcare system, including adaptation measures of infra-structures and logistics to major climate-related events that also mitigate emissions, strengthening health surveillance systems for early detection of climate-sensitive diseases and conditions, training activities for health professionals and providing timely science-based information to the Unified Health System (SUS) managers. Social participation in the decision-making process of the SUS is mandatory by law in Brazil, which can leverage support for the health sector’s adaptation and mitigation plan and public policies with the best climate and health cobenefits.

Kevin Cromar of the NYU Marron Institute discussed practical governmental policy-related activities to better promote both health and climate action. One important pathway identified was improvement as to how health impacts are considered in economic models applied to evaluate government policy options. The social cost of carbon dioxide (SC-CO_2_) is the monetized value of the damages to society per ton of CO_2_ emissions in cost-benefit analysis. These SC-CO_2_ estimates rely on climate science, economics, demography, and other disciplines. Recent higher SC-CO_2_ values, versus past estimates, substantially increase the estimated benefits of greenhouse gas mitigation, thereby increasing the expected net benefits of stronger climate policies.^28,29^ These economic analyses advance allow for a fuller characterization of costs, including sectoral market and nonmarket damages to human health, helping decision-makers optimize health in policy decision-making.

Oriol Teixidó from Ricardo plc. emphasized the importance to bridge the gap between research and government action. Translating identified solutions into policy actions requires: (i) laying the groundwork, (ii) gathering the evidence and building support for the approach, (iii) identifying the policy windows where solutions can be incorporated and implemented, and (iv) presenting the evidence in a way that is useful for decision-makers and specific to the policy window identified. An international example of a successful translation of the latest scientific research to support climate policy is the Climate Services for a Net Zero Resilient World (CS-NOW), a £5 million research program funded by the UK Department for Energy Security and Net Zero (https://www.gov.uk/government/collections/climate-services-for-a-net-zero-resilient-world).

Zorana Andersen of the University of Copenhagen discussed the past and potential roles of scientific societies in promoting greater consideration of environmental health in policy decisions. Examples included:

- ISEE and American Thoracic Society (ATS) Policy Committee editorials, such as with other medical societies in support of WHO 2021 Air Quality Guidelines (see https://www.iseepi.org/policy_and_advocacy_statements.php);
- Writing to/meeting with governmental representatives, such as EU Ministers, and with representative of the Office of Management and Budget in the United States;
- Participation in government hearings, such as on the EU Ambient Air Quality Directive (AAQD) Proposal (see https://www.europarl.europa.eu/legislative-train/theme-a-european-green-deal/file-revision-of-eu-ambient-air-quality-legislation), and US EPA air quality standard setting hearings;
- Serving as expert advisor on government panels, such as on the US EPA Clean Air Science Advisory Council (CASAC) panels, and the US EPA Board of Scientific Counselors (https://www.epa.gov/bosc);
- Organizing environmental health press conferences, such as the “Clean Air in Europe For All” –Brussels meeting, 24 May 2023.^30^

The ISEE’s Policy and Advocacy Statements can be found online at https://www.iseepi.org/policy_and_advocacy_statements.php. Similarly, the ISEE’s US Chapter’s Policy Committee submissions to the EPA and other governmental agencies can be found online at https://isee-northamerica.github.io/isee-nac/policy.html.

### Future opportunities for public health engagement

During the second day of the ISEE Workshop, discussions of day 1 presentations sought to identify culturally appropriate ways that health professionals, planners, decision-makers, and patient organizations can help implement these health policy pathways that are also climate and sustainability friendly. Based on the above-noted materials, the group recommended these civic engagement actions to public health and healthcare professionals:

- Participate in policy considerations (e.g., government advisory committee hearings, such as to the US EPA) to support healthy policies that also mitigate climate change, such as via stronger environmental controls;
- Host and participate in public forums on the human health benefits of cleaner air;
- Advise governments to implement more sustainable and healthier public food procurement guidelines, such as to the US Congress and the European Union Commission;
- Reduce the health and climate impacts of transportation by endorsing, at city government hearings, improved and subsidized public transport, improved active travel infrastructure, compact cities, and cleaner, quieter low carbon vehicles;
- Support local combustion-free buildings, including reducing the prevalence of fossil fuel gas homes and businesses at local, city, or state government venues;
- Promote the use of lower climate-forcing medications in healthcare; and
- Advocate at our workplaces for reduced carbon footprints in the operation of health and academic employment institutions.

Unlike many proposed climate-forcing emissions control measures, public health and urban planning policies already have existing regulations and policy guidelines that can be readily implemented to achieve both public health and associated climate mitigation progress. However, when following these recommendations, it is important we start with health messages that are readily understandable, to most efficiently accelerate the transition to a net zero emission target and create conditions for the significant policy changes that are highly and urgently needed.

## Conflicts of interest statement

The authors declare that they have no conflicts of interest with regard to the content of this report.

## Acknowledgments

This Workshop Report was prepared by an ad hoc committee of the International Society for Environmental Epidemiology.

## Supplementary Material


