# Supplementary figures and images for: Transitioning from climate ambitions to climate actions through public health policy initiatives
Source: Environ Epidemiol. 2025 Mar 6;9(2):e373. doi: 10.1097/EE9.0000000000000373 (PMC11888974; doi:10.1097/EE9.0000000000000373)

Figure S1. ISEE Policy Summary of Health Co-Benefits of Climate Action (Roca-Barceló et al, 2024)

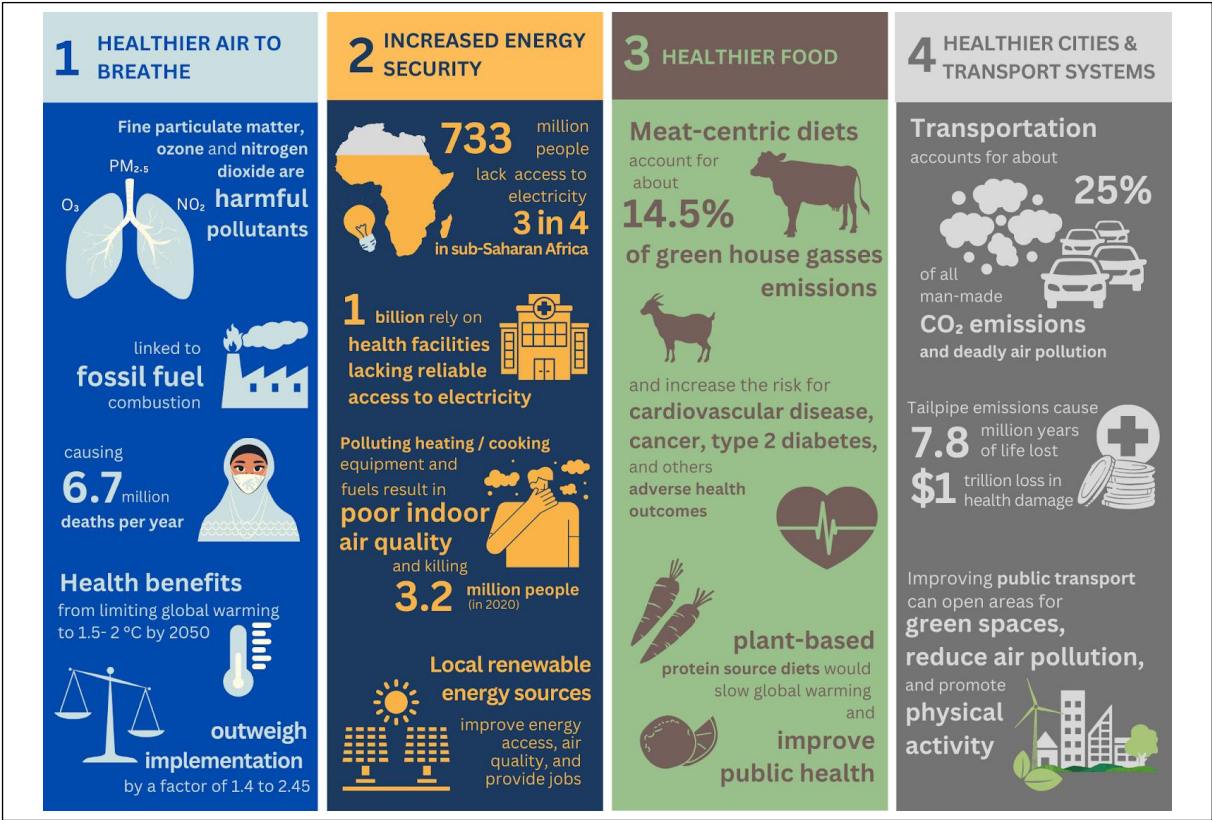

Supplement: Supplementary file 1 [file ee9-9-e373-s001.pdf]
